# Detection of Geometric Risk Factors Affecting Head-On Collisions through Multiple Logistic Regression: Improving Two-Way Rural Road Design via 2+1 Road Adaptation

**DOI:** 10.3390/ijerph18126598

**Published:** 2021-06-19

**Authors:** Laura Cáceres, Miguel A. Fernández, Alfonso Gordaliza, Aquilino Molinero

**Affiliations:** 1 Departamento de Estadística e Investigación Operativa, Escuela de Ingenierías Industriales, Universidad de Valladolid, 47011 Valladolid, Spain; lauradcr23@gmail.com (L.C.); alfonsog@eio.uva.es (A.G.); 2 Escuela de Ingenierías Industriales, Universidad de Valladolid, 47011 Valladolid, Spain; aquilino.molinero@alumnos.uva.es

**Keywords:** road safety, head-on crash, two-way rural roads, centerline treatment, 2+1 roads

## Abstract

This study aims to characterize locations on two-way rural roads where head-on crashes are more likely to occur, attending to geometric road design factors. For this purpose, a case-control study was carried out using multiple logistic regression models with variables related to road design parameters, considering several scenarios. The dataset corresponding to cases (places where crashes have occurred) was collected on Spanish “1+1” rural roads over a four-year period. The controls (places where no crashes have occurred in the period) where randomly selected through a specific ad hoc designed method. The obtained model identifies risk factors and allows the computation of the odds of a head-on collision on any specific road section: width of the pavement (when it exceeds 6 m), width of the lanes (for intermediate widths between 3.25 and 3.75 m) and tight curves (less than 250 m of radius) are identified as factors significantly increasing the odds of a crash, whereas a paved shoulder is a protective factor. The identified configurations on two-way rural roads may be susceptible to transformation into “2+1” roads to decrease the odds of a head-on crash, thus preventing possible serious injuries and enhancing transportation safety.

## 1. Introduction

In most countries around the world, the highest percentages of all road fatalities come from crashes on rural roads (for instance, in the European Union -EU-, fatalities on these roads represent 55% of the total number [1]). Among these interurban roads, the most frequent type is the two-way rural road with only one carriageway, in particular, the so-called “1+1” roads (two-way rural roads, with one lane for each direction in the same carriageway and where overtaking is possible). For instance, they represent around 65% in the USA [2] or 80% in Spain [3], of overall road network kilometers, and they are where the highest percentage of fatalities on rural roads happen (75% in Spain [3], 90% in the USA [4]), even though traffic density on these roads is lower than on motorways.

**Figure 1:**
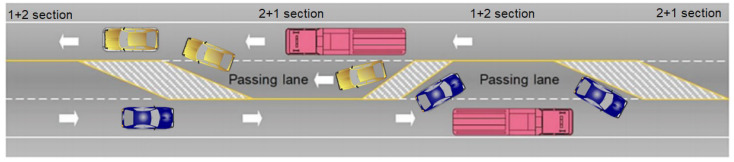
Example of 2+1 road section in a two-way interurban road with only one carriageway.

Studies [3] indicate that the frequency of head-on crashes, especially fatal head-on crashes, is much higher on undivided rural two-lane highways than other types of roadways. On these two-lane roads, vehicles crossing the centerline of two-lane roads and striking opposing vehicles head-on account for 20% of all fatal crashes [5]. One possible solution to avoid head-on crashes and prevent the associated injuries is to transform these “1+1” roads into the so-called “2+1” roads which are two-way rural roads, with one or two lanes in each direction in the same carriageway and where both ways are separated through the installation of a road restraint system or any device to avoid the presence of a vehicle in the opposite direction (see Figure 1). This type of road is used as an intermediate solution between the common two-lane road and a dual carriageway road.

Successful experiences concerning this transformation have been reported in different countries, such as Germany (“2+1” roads have been found to operate with crash rates 36% lower than conventional two-lane highways), Sweden (55% lower), Finland (22–46% lower), Poland, or Texas (US). This suggests that these transformations are potentially applicable in other countries. This is the reason for the increasing interest in “2+1” roads in such countries as the USA, Canada, Australia, New Zealand, Poland, Mexico or Spain [6,7].

There is extensive literature on rural head-on crashes. Table 1 contains a non-exhaustive collection of contributions and findings. Some of these contributions come from descriptive studies [8,9], while others come from inferential ones, using different models and schemes [10,11,12,13,14,15,16,17,18,19,20,21,22,23,24]. However, there are not so many results concerning “2+1” roads and they are mainly descriptive [25,26,27,28]. See also Table 1 for the main results obtained in these studies.

In this study, we consider an inferential case-control framework and, using head-on crash data from a four year period, we analyze and determine which geometric road factors have a significant influence on the odds of the appearance of these crashes in a rural road section. The influence of each factor is quantified using a multiple logistic regression model. The model obtained can help road managers to prioritize their resources when deciding which sections of “1+1” roads are more in need of transformation into a “2+1” road to decrease the odds of a head-on crash and its associated injuries.

## 2. Materials and Methods

### 2.1. Data

The study analyzes crash data collected from national and regional roads in the Spanish region of Castilla y León over the most recent four-year period with available data. Castilla y León is the largest and sixth most populated Spanish region and can be considered representative of many other regions in Spain.

To carry out this study, the so-called “ARENA” database was used. This database is designed and maintained by the Spanish General Directorate of Traffic and contains information from all injury traffic crashes on Spanish roads. The information in ARENA is gathered by the Spanish national traffic police by asking people involved in the crash and carrying out the respective reconstructions. These data are fully reliable, as shown by the fact that they are used in trials related to traffic crashes.

Some of the variables included in ARENA and considered in this study correspond to the geometric design parameters of the road where the crash occurred. Two additional variables are also considered here: “Radius of curvature”, obtained using measurement tools available on Google Maps (maps.google.es, accessed on 1 May 2021), and “Average daily traffic” (ADT), obtained from official sources [29,30].

### 2.2. Variables

The set of variables included in this study are shown in Table 2 (Variables V1 to V9) and were selected because they are geometric road design parameters (except the variable ADT, which usually has a role in crash appearance). In our study, continuous variables were transformed into categorical variables using objective categorization criteria [31]. This allowed for the easy consideration of non-linear relationships (such as the one appearing in variable V2) between these variables and the outcome. Several possibilities were considered for this categorization and it was found that no significant differences appeared due to different categorizations.

No variables related to speed are included in the study because the speed limit considered on the two-way rural roads is constant (90 Km/h). Notice that, as explained below, the individuals in our study are the locations (characterized by geometric parameters of road design) where crashes have occurred, not the crashes themselves.

### 2.3. Study Design and Sample Selection

A case-control design has been used to identify road-related risk factors associated to locations where head-on crashes can occur. According to [24], this framework is better than other epidemiological designs (as, for instance, cohort designs) to estimate the magnitude of the association between the different exposures considered, when the outcome appears in a small fraction of the exposed and unexposed individuals. The case-control design used is retrospective, which means that what is known at the beginning of the study is the presence/absence of the outcome and then the possible previous exposure is investigated. The outcome considered was the occurrence of a head-on crash in either a straight section or on a curve on two-way rural roads (“1+1” roads) which could easily be transformed to “2+1” roads and so avoid head-on crashes. It is important to notice that the individuals in our study are the locations where crashes have occurred (cases) or where they have not (controls), not the crashes themselves; we can therefore use the term “sites” when we refer to the “individuals” in the study.

The following crashes have been considered to select the cases: head-on and side impact collisions on two-way rural roads belonging either to the Spanish National or Regional road networks in the region of Castilla y León, which occurred on “1+1” road sections without any intersections. Crashes that occurred in rural roads belonging to local/provincial administrators have not been considered, since these roads are usually much narrower than the ones belonging to national and regional networks. This narrowness makes the eventual transformation of these roads into “2+1” roads unlikely because it would require an important amount of resources to afford the changes, which are not usually available to local/provincial administrations. The inclusion of side impact collisions (T-bone crashes) in the study is because they are close to head-on collisions in road sections without intersections (the driver tries to overtake, as during head-on crashes, but the avoiding maneuver means the driver turns the steering wheel abruptly). These head-on and side impact collisions represent 588 cases, 310 on straights and 278 on curves. The study covers all types of vehicles in the proportions that they were involved in the crashes: at least one passenger car was involved in 90% of these cases, a van or truck in 18%, a motorcycle in 11%, a bicycle in 2% and a coach in 1%.

Taking into account the available number of cases, the use of a ratio of controls to cases 1:1 is an adequate strategy. In fact, using simple logistic regression models, the sample size available (*n* = 588 cases in the “straigths+curves” scenario) would allow a very high power (above 90%) to be achieved at the usual significance level of 5% to detect raw odds-ratios rOR ≥ 1.5 for a hypothetical proportion of 0.3 for exposed controls. In the case of a hypothesized proportion of 0.2 for exposed controls, the achieved power would be higher than 80%. In the separate “straights” or “curves” scenarios, the mentioned powers would be achieved to detect rOR ≥ 2.

**Figure 2:**
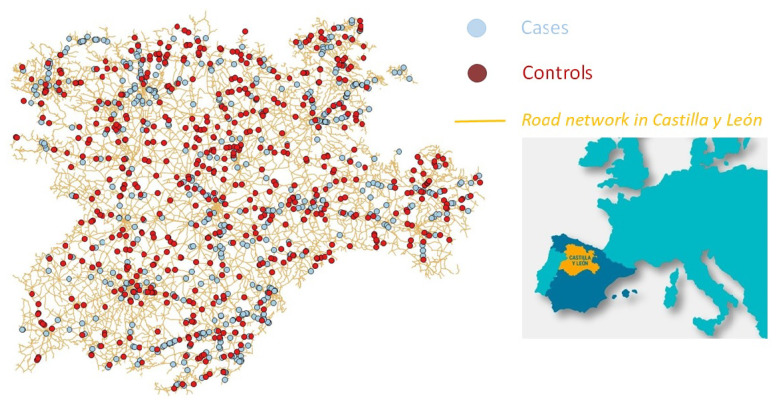
Location of the cases and controls in the road network in the region of Castilla y León.

The controls were randomly selected from all the population individuals (sites) that did not suffer the said outcome in that period. This random selection is not an easy task, due to the inexistence of an organized census containing all straights and curves in the roads of the region of Castilla y León. To perform a truly random selection, the map of roads in this region was used to divide all the roads belonging to the two networks previously mentioned into small one hectometer sections. These sections were then ordered and numbered, resulting in a total of 164,596 hectometers of this type of roads. Then a discrete uniform distribution on values {0,⋯,164,596} was considered to select the sample of controls, extracting 588 values of the distribution. They were then transformed into the corresponding road+hectometer section. If any of these values corresponded to a road section where a head-on crash had happened (cases), then a new value from the uniform distribution was selected. The values of each variable in these locations were obtained using the software Iberpix 4 (http://ign.es/iberpix2/visor/, accessed on 1 May 2021) and Google Maps (maps.google.es, accessed on 1 May 2021), instead of visiting them in person. Figure 2 shows the location of the cases and controls considered in this study and how the controls are spread over the road network.

As is well known, the selection of controls is a key concern in case-control designs. Selection bias is a threat that usually hangs over case-control designs, causing the lack of comparability between the two groups studied. The selection scheme followed in this study, based on a random selection of the road network, is a completely impartial mechanism that favors the non-appearance of this bias.

### 2.4. Analysis

As noticed by [32], “logistic regression is a popular statistical modeling method to find relationships between accident variables and the outcome” (see [33,34,35,36,37]). In this study we consider it to analyze the data for three different situations: curves, straights, and the combination of both. The reason for studying these three scenarios is that, in other studies [38], the significant variables for curves are different from those of the other scenarios. For each of these situations, unadjusted logistic regression models were used to compute rOR to check which road-related variables may influence the outcome. Multiple logistic regression, including all possible explanatory variables, served to calculate the adjusted odds-ratios (aOR) that show the role of each variable in the presence of the others in the model. Stepwise selection procedures (backward and forward) were considered to determine simpler logistic regression models. From these procedures, two models for each of the three abovementioned situations were selected: a full model including all variables and the model resulting from a backward elimination (which, in all three cases, coincided with the model obtained using forward selection). In order to select a final model for each scenario, we considered two criteria: the proportion of correctly classified observations (using a leave-one-out -LOO- cross validation procedure to avoid upwards bias) and the area under the ROC curves (AUC) [39] of each model compared using the DeLong Test [40]. AUC was chosen instead of other criterion, such as the Akaike Information Criterion (AIC), as AUC is more closely related to a good classification performance than AIC, which mainly deals with model fitting. All these analyses were performed with IBM SPSS software (Armonk, NY, USA) for model estimation and variable selection, and SAS software (Cary, NC, USA) for the LOO evaluation and ROC curves tests.

## 3. Results

### 3.1. Descriptive Analysis

Table 2 shows the frequency distribution (in percentages) of all the variables considered in the study for both controls and cases.

### 3.2. Analytical Study

The rOR, provided in Table 2, were obtained by studying the effect of each single predictor variable in the logistic regression models. It is important to note that almost all the variables are significant in the rOR analysis and that the values obtained are coherent with those appearing in the subsequent aOR analysis. Perhaps the most surprising finding is that the presence of reflective edge posts (V6) seems to significantly increase the crash risk. Notice that we are not claiming that the posts are a cause for the crashes as, for example, they may have been installed in locations identified as problematic (and not been enough to solve the problem).

As for the multiple logistic regression models, Table 3 shows the results corresponding to the analysis of the ROC curves and the LOO values for the two models considered for the three situations (straights + curves, straights only and curves only). In all three cases, the backward model was chosen over the full model since the backward model is simpler, the LOO values are similar (even higher for the backward in the only curves scenario) and there was no significant difference in the AUC values using the DeLong test.

Table 4 shows, for the chosen models and for each one of the three scenarios, which variables and values increase the odds of a head-on crash on a two-way rural road (risk factor) or decrease it (protective factor). The goodness of fit for these regressions is reasonable, as suggested by the Hosmer-Lemeshow (HL) goodness of fit tests, also given in Table 4. It is interesting to notice that the models obtained for the three scenarios are similar. The same variables are selected for the curves only and the joint curves and straights scenarios. Moreover, since V3 (radius of curvature) makes no sense for the only straights scenario, the only difference between this scenario and the other two is that the reflective edge posts variable (V6) does not appear in the straights only model. For these reasons, we focus our conclusions on the joint straights + curves scenario.

Table 4 also shows that, for this joint scenario, the width of the pavement (V1) is a significant risk factor, with an aOR of 13.684 for roads with pavement wider than 7 m. The odds of a head-on crash on a road where the pavement is wider than 7 m is more than 13 times higher than that of a head-on crash on a road where the pavement is less than 6 m wide. However, the influence of the width of the traffic lane (V2) is not linear, since lanes between 3.25 and 3.75 m show increased odds over those less than 3.25 m wide (aOR = 1.605), while the odds for those wider than 3.75 m are lower, with an aOR of 0.339. The results for radius of curvature (V3) show that the risk of a curve section over a straight one only increases significantly if the curve is tight, with an aOR of 3.449 for curves with less than 250 m of radius. As for the width of the shoulder (V4), the existence of a practicable shoulder is a protective factor, with protection increasing with the width of the shoulder, with an aOR of 0.032 for paved shoulders more than 2.5 m wide. This means that the odds of a crash in this situation is more than 31 times lower (1/0.032 = 31.25) than those in a road section with a nonexistent or impractical shoulder. The influence of reflective edge posts (V6) is, perhaps surprisingly, negative; as its presence increases the odds of a crash, especially in curves, since the variable was not significant in straight road sections. Finally, the average daily traffic (V8) was significant, as expected, with higher values of the variable leading to higher crash odds.

Notice that other factors, such as side safety barriers (V5), sidewalk (V7) and number of lanes (V9), do not appear in this model, as the outcome variability they explained in the initial simple models is accounted for by other variables in the final multiple models.

## 4. Discussion

There are several studies in the literature dealing with head-on crashes on rural roads and also some considering the characteristics of “2+1” roads and how these help to reduce crashes. However, many of these studies are not inferential as the one performed here, but descriptive [8,9,25,26,27,28], so no statistical inference can be obtained, i.e., their results should not be extrapolated. Among the inferential studies, some of them consider all types of crashes [10,11,12,13,14,15,16,17,18,19,20,21,22,23,24], which is not convenient to our purpose, as we are studying the crashes that can be avoided by “2+1” road transformation. Other interesting studies consider non geometric risk factors, such as driver behavior [10] or environmental conditions [12]. The studies considering geometric risk factors sometimes detect factors similar to those found here, but under different settings. For example, [12] detects a higher collision risk on roads 7 m wide or wider, but considers the likelihood of head-on crashes vs. other types of crashes not against the likelihood of no crash, as done here. Another example can be found in [11], where horizontal curvature is found to increase crash frequency and the presence of a shoulder to decrease it, but in that study, two way interurban roads with two carriageways are also considered, which is not our case, as those roads cannot be transformed to “2+1” roads. In this sense, although there is no big surprise in the risk factors detected here, this study is useful as it has been specifically designed to account for the geometric road factors affecting head-on collisions that can be considered when deciding which road sections are to be transformed into “2+1” roads.

There are two main contributions in this study compared to what is already known. The first is a methodological contribution, i.e., the control selection method developed in Section 2.3 which is intended to avoid selection bias. The second is the determination, through an inferential case-control study, of which geometric road design factors are significantly influential on the odds of a head-on crash appearing in a rural road section and the extent of this influence. From the results explained in the previous section, we can determine which sections of “1+1” roads are more in need of transformation into a “2+1” road to decrease the odds of a head-on crash, and these results will then be useful for road administrators, optimizing their resources to obtain the highest possible crash and injury reduction.

For example, if we consider the design characteristics only, the sections with the highest crash odds are those with a wider pavement (more than 7 m) and an intermediate width of the traffic lane (between 3.25 and 3.75 m), a nonexistent or impractical shoulder, the presence of reflective edge posts, and containing a tight curve (with less than 250 m radius). A road section with all these characteristics suffers from crash odds more than 170 times higher (exp (2.616 + 0.473 + 1.238 + 0.810) = 170.204) than a section without any of them.

One of the risk factors detected is a pavement width greater than 7 m. Notice that this is not bad, as it will obviously be cheaper to implement a third lane (transformation from “1+1” to “2+1”) because the pavement is wide. Together with the width of the pavement, the width of the traffic lane should also be considered, since intermediate values of this variable seem to produce a false feeling of security in drivers, leading to higher odds of a crash. Road sections without a practical shoulder should also be considered as candidates to implement a third lane, since the presence of a wide shoulder is a protective factor.

It is also convenient to notice that, according to the results, there is no significant advantage obtained from acting on wide or medium curves, as no significant crash odds reduction is achieved if the radius is not less than 250 m. This is also an interesting conclusion, from the practical point of view, since curve sections are more likely to result in higher costs when implementing a third lane.

Another point to mention is that, although ADT is not a variable related to the geometric design of the road, it should clearly be considered for prioritization among road sections with similar characteristics, as higher values of this variable are significantly related to higher crash odds.

### Limitations and Future Work

Although some other Spanish regions [41] are interested in the transformation of “1+1” roads to “2+1” roads, the study focused only on the region of Castilla y León. It would be interesting to extend it to other Spanish regions to increase the number of cases-controls (sample size), and thus strengthen the representativeness of the results obtained.

We would also have liked to have more recent data for our study. However, in Spain, these data are made available with a long-time delay. The most recent “Main figures on Road Traffic Accidents in Spain” [3] are from 2019 and contain data from 2018. However, for a study like this, we need the microdata and the most recent microdata available are from 2015. Nevertheless, although our data are not as recent as we would like, they are still consistent with today’s conditions. In the previously mentioned report, we can see that the variation in the number of deaths or injured people in traffic crashes is about 10% and has come (after many years) to some sort of stability.

## 5. Conclusions

One way of preventing head-on crashes on “1+1” rural roads is to transform them into “2+1” roads and avoid dangerous overtaking without harming the volume of traffic. These “2+1” roads are receiving increasing attention in the literature and there are some interesting studies concerning their characteristics, the volume of traffic they may support and how the overtaking sections should be designed [13,14,25,26,27,28].

There is already also some literature studying which geometric road factors are related to head-on crashes in two-way rural roads, but there are no inferential studies focused specifically on detecting “1+1” locations susceptible to be transformed into “2+1” roads, due to their specific geometric configuration. Using a statistical inferential data analysis under a case-control framework, this article has focused on risk assessment and road safety management, detailing the geometric risk factors characterizing these locations. In this way, we have quantified the influence on the risk of each geometric road factor considered and found that the absence of a paved shoulder, the width of the pavement, an intermediate traffic lane width and the presence of a tight curve, increase the odds of a crash. Other factors such as side safety barriers or sidewalks seem to have a marginal influence when the previous factors are accounted for. While these results may not be surprising, the quantification of the influence of the significant factors obtained here should allow road managers to compute the crash odds of any particular road segment and thus to prioritize which road segments should be taken care of first.

We believe that the results set out here, together with those on the design of overtaking sections that appear in other studies such as [13,14], can also be considered for future well-designed and properly maintained roads, with the aim of preventing these head-on crashes. In fact, some current directives, guides, or recommendations [7,42,43,44] are already focusing on comprehensive transportation safety aspects of infrastructure design that must be considered to avoid head-on crashes.

## Figures and Tables

**Table 1:** Literature review summary.

Scope	Ref.	Type of Study	Geometric Road Factors or Other Findings Detected
Rural head-on crash rates	[8]	Descriptive	Rural head-on crashes happen in the following situations: Poor sight distance for overtaking due to horizontal and vertical curves; frequent horizontal or vertical curves; poor curve delineation; unsealed or partially sealed shoulders; insufficient or unclear advisory/warning signs; insufficient or poorly maintained raised reflective pavement markers; areas subject to fog.
Rural head-on crashes on two-way roads	[9]	Descriptive	Drivers most commonly lost control of their vehicles by entering right-hand curves, which is likely to be influenced by the radius of the curve, the distance from the previous curve, and the roadway width. Other causes include over-correction after running off the right edge of the pavement, which may be affected by the design and quality of the pavement edge.
	[10]	Inferential	Considers five different types of crash (not only head-on). Identifies (logistic regressions) road construction and behavioral risk factors for fatal vs. slight injuries.
	[11]	Inferential	Road geometric characteristics (using random-effect negative binomial, RENB, models) of head-on crashes frequency in rural and semi-urban areas (two-way interurban roads with one or two carriageways, instead of only two-way interurban roads with only one carriageway): horizontal curvature, terrain type, and access points were found to be positively related to the frequency of head-on crashes, while larger shoulder width decreased the crash frequency.
Rural head-on crashes on Spanish two-way roads	[12]	Inferential	Identifies (using multivariate robust Poisson regression model) the road factors associated with the likelihood of head-on crash with respect to other types of crash (instead of the likelihood of head-on crashes with no crashes) having happened on two-way interurban roads with one or two carriageways: More likely on wide roads, on road sections with curves, narrowings or drop changes, on wet or snowy surfaces, and in twilight conditions. Lower probability with the existence of medians and a paved shoulder.
	[13,14]	Inferential	Define (using methodologies such as the highway capacity manual, HCM, from the USA Transportation Research Board) the lengths of the zones where overtaking could be safe, avoiding head-on collisions on 1+1 two-way rural roads (does not consider risk factors for head-on collisions).
	[15]	Inferential	Quantifies (using HCM methodology) the performance of two lane highways (does not consider risk factors for collisions).
Rural crashes on two-way roads	[16,17,18,19,20,21,22,23,24]	Inferential	These papers consider different research designs on any type of crash: Potential-for-Crash Reduction Method [16]. Sites-With-Promise (SWiP) [17]. Method retrospective cohort studies; collision-based observational Before-After Studies [18]. Random parameters multivariate Tobit (RPMV-Tobit) models [19]. Random parameters bivariate ordered probit models [20,21]. Univariate Poisson models [22]. Bayesian network approach [23]. Case-control design [24].
2+1 roads	[25,26,27,28]	Descriptive	2+1 roads are effective in the following situations: For higher-volumes or in areas where minor intersections and driveways provide direct access to the roadway. Also, in mountainous terrain with long, steep grades. 2+1 road design focuses on: cross section, lane and shoulder widths, alignment, traffic flow, transition zone length, separation of opposing lanes, intersections and access control and marking and signing.

**Table 2:** Raw odds-ratios (rOR) and their 95% CIs for the variables considered in each of the three different scenarios, where * stands for *p*-value ≤0.1, ** for *p*-value ≤0.05 and *** for *p*-value ≤0.01. Sample size n=588 (310 straights and 278 curves) was used for both cases and controls.

Description		Proportion		Raw Odds-Ratios (rOR)		
Variable	Category	Cases	Controls	Straights + Curves	Straights	Curves
Width of pavement (V1)	<6 m (ref)	6.7	33.8			
	6–7 m	29.4	38.3	3.923 ** (2.640, 5.831)	4.836 ** (2.661, 8.788)	3.471 ** (1.991, 6.052)
	>7 m	63.9	27.9	11.699 ** (7.927, 17.264)	16.000 ** (8.941, 28.633)	9.444 ** (5.406,16.497)
Width of traffic lane (V2)	<3.25 m (ref)	24.7	61.7			
	3.25–3.75 m	72.6	35.9	5.066 ** (3.932, 6.528)	6.184 ** (4.371, 8.749)	4.726 ** (3.161, 7.067)
	>3.75 m	2.7	2.4	2.861 ** (1.361, 6.013)	6.421 * (1.493, 27.614)	1.524 (0.632, 3.673)
Radius of curvature (V3)	Straight (ref)	52.7	66.3			
	Wide radius (>750 m)	3.6	2.9	1.554 (0.806, 2.997)	-	(ref)
	Medium radius (750–250 m)	16.1	12.2	1.660 * (1.181, 2.334)	-	1.068 (0.526, 2.170)
	Tight radius (<250 m)	27.6	18.6	1.870 ** (1.406, 2.486)	-	1.203 (0.607, 2.384)
Width of shoulder (V4)	None-impractical (ref)	17.2	25.8			
	<1.5 m (paved)	59.7	54.4	1.651 ** (1.231, 2.214)	2.118 ** (1.407, 3.188)	1.217 (0.778, 1.904)
	1.5–2.5 m (paved)	21.6	17.3	1.874 ** (1.304, 2.692)	1.344 (0.426, 4.242)	1.305 (0.700, 2.432)
	>2.5 m (paved)	1.53	2.4	0.967 (0.404, 2.319)	1.344 (0.426, 4.242)	0.662 (0.168, 2.604)
Side safety barrier (V5)	No (ref)	58.2	74.3			
	Yes	41.9	25.7	2.082 ** (1.626, 2.665)	2.071 ** (1.431, 2.996)	1.627** (1.127, 2.349)
Reflective posts (V6)	No (ref)	36.2	68.7			
	Yes	63.8	31.3	3.866 *** (3.034, 4.925)	3.307 *** (2.421, 4.515)	5.105 *** (3.423, 7.614)
Sidewalk (V7)	No (ref)	96.3	97.8			
	Yes	3.7	2.2	1.719 (0.858, 3.446)	2.304 ** (1.004, 5.287)	1.070 (0.298, 3.842)
ADT (V8)	<500 (ref)	9.7	35.9			
	500 ≤ ADT < 2000	33.2	44.6	2.755 *** (1.949, 3.895)	3.422 *** (2.048, 5.716)	2.488 *** (1.515, 4.085)
	2000 ≤ ADT < 10,000	51.8	18.9	10.138 *** (7.042, 14.597)	14.050 *** (8.303, 23.775)	8.006 *** (4.648, 13.789)
	≥10,000	5.3	0.6	29.201 *** (9.727, 84.615)	28.424 *** (7.548, 107.039)	37.400 *** (4.786, 292.263)
Number of lanes (V9)	1+1 (ref)	94.4	98.1			
	1+n or n+1 (n > 1)	5.6	1.9	3.119 *** (1.561, 6.232)	2.546 *** (0.632, 10.263)	2.555 ** (1.135, 5.750)

**Table 3:** Model selection for the three scenarios (results from Backward and Forward selection model were the same).

Data	Model	Variables in Model	LOO	AUC	*p*-Value
Scenario “Straights + Curves”	Full	All	74%	0.8306	0.4631
	Backward	V1, V2, V3, V4, V6, V8	73.9%	0.8295	
Scenario “Only Straights”	Full	All (except V3)	73.8%	0.8167	0.6572
	Backward	V1, V2, V4, V8	73.1%	0.8147	
Scenario “Only Curves”	Full	All	76.5%	0.8541	0.3354
	Backward	V1, V2, V3, V4, V6, V8	77.1%	0.8519	

**Table 4:** Final models selected for each scenario, where ** stands for *p*-value ≤0.05 and *** for *p*-value ≤0.01.

		Straights and Curves		Straights Only		Curves Only	
Variable	Category	ß	Exp (ß)- aOR 95% CI	ß	Exp (ß)- aOR 95% CI	ß	Exp (ß)- aOR (95% CI)
Constant		−2.226 ***	0.108	−2.303 ***	0.100	−2.367 ***	0.094
Width of pavement (V1)	<6 m (ref)						
	6–7 m	1.891 ***	6.626 (3.643, 12.052)	1.537 ***	4.651 (2.126, 10.174)	2.667 ***	14.398 (4.910, 42.222)
	>7 m	2.616 ***	13.684 (6.604, 28.353)	2.451 ***	11.603 (4.625, 29.106)	3.438 ***	31.118 (8.378, 115.572)
Width of traffic lane (V2)	<3.25 m (ref)						
	3.25–3.75 m	0.473 **	1.605 (1.030, 2.501)	0.643 **	1.902 (1.085, 3.334)	0.034	1.034 (0.488, 2.192)
	>3.75 m	−1.082 **	0.339 (0.124, 1.030)	−0.209	0.811 (0.153, 4.307)	−1.974 ***	0.139 (0.033, 0.581
Radius of curvature (V3)	Straight (ref)			-	-		
	Wide radius (>750 m)	0.077	1.080 (0.483, 2.415)	-	-	(ref)	
	Medium radius (750–250 m)	0.161	1.174 (0.783, 1.761)	-	-	0.157	1.170 (0.471, 2.908)
	Tight radius (<250 m)	1.238 ***	3.449 (2.361, 5.038)	-	-	1.421 ***	4.140 (1.576, 10.8787)
Width of shoulder (V4)	None-impr. (ref)						
	<1.5 m (paved)	−1.628 ***	0.196 (0.116, 0.333)	−1.052 ***	0.349 (0.179, 0.680)	−2.500 ***	0.082 (0.031, 0.218)
	1.5–2.5 m (paved)	−2.737 ***	0.065 (0.034, 0.124)	−2.130 ***	0.119 (0.054, 0.262)	−3.451 ***	0.032 (0.010, 0.105)
	>2.5 m (paved)	−3.455 ***	0.032 (0.10, 0.095)	−2.750 ***	0.064 (0.016, 0.249)	−4.372 ***	0.013 (0.002, 0.087)
Reflective posts (V6)	No (ref)			-	-		
	Yes	0.810 ***	2.247 (1.591, 3.174)	-	-	1.564 ***	4.776 (2.665, 8.559)
ADT (V8)	<500 (ref)						
	500 ≤ ADT < 2000	0.646 ***	1.907 (1.257, 2.894)	0.789 ***	2.200 (1.238, 3.912)	0.497	1.643 (0.870, 3.103)
	2000 ≤ ADT < 10,000	1.789 ***	5.983 (3.710, 9.467)	1.896 ***	6.660 (3.493, 12.699)	1.861 ***	6.429 (3.027, 13.657)
	≥10,000	2.947 ***	19.053 (5.868, 61.865)	2.860 ***	17.467 (4.210, 72.465)	3.251 ***	25.818 (2.749, 242.502)
**Hosmer-Lemershow GoF test**		$\chi^{2}$ = 2.386, df = 8, *p*-val = 0.967		$\chi^{2}$ = 3.055, df = 8, *p*-val = 0.931		$\chi^{2}$ = 11.284, df = 7, *p*-val = 0.127	

## Data Availability

The data used in this study are available on request from the corresponding author.

## Abbreviations

The following abbreviations are used in this manuscript:

ADT	Average Daily Traffic
rOR	Raw Odds Ratio
aOR	Adjusted Odds Ratio
LOO	Leave One Out
AUC	Area Under ROC Curve
ROC	Receiver Operating Characteristic
CI	Confidence Interval
HL	Hosmer-Lemershow
GoF	Goodness of Fit
